# Supplementary material for: In situ inorganic conductive network formation in high-voltage single-crystal Ni-rich cathodes
Source: Nat Commun. 2021 Sep 7;12:5320. doi: 10.1038/s41467-021-25611-6 (PMC8423756; doi:10.1038/s41467-021-25611-6)
Supplement: Supplementary file 1 — Supplementary information [file 41467_2021_25611_MOESM1_ESM.pdf]

Supplementary Information

In Situ Inorganic Conductive Network Formation in High-Voltage Single-Crystal Ni-rich Cathodes

Xinming Fan[1], Xing Ou[1], Wengao Zhao[2, 3]*, Yun Liu[1], Bao Zhang[1], Jiafeng Zhang[1], Lianfeng Zou[4], Lukas Seidl[2], Yangzhong Li[5], Guorong Hu[1], Corsin Battaglia[2], Yong Yang[3]**

1. X. Fan, Dr. X. Ou, Y. Liu, Prof. B. Zhang, Dr. J. Zhang, Prof. G. Hu
School of Metallurgy and Environment, Central South University, Changsha 410083, P.R. China, E-mail: ouxing@csu.edu.cn

2. Dr. W. Zhao, Dr. L. Seidl, Dr. C. Battaglia
Empa, Swiss Federal Laboratories for Materials Science and Technology, 8600 Dübendorf, Switzerland, E-mail: wengao.zhao@empa.ch

3. Dr. W. Zhao, Prof. Dr. Y. Yang
School of Energy Research, Xiamen University, Xiamen, Fujian 361005, P.R. China,
E-mail: yyang@xmu.edu.cn

4. Dr. L. Zou
Environmental Molecular Sciences Laboratory, Pacific Northwest National Laboratory, Richland, Washington 99354, United States

5. Dr. Y. Li
High Performance Computing Department, National Supercomputing Center in Shenzhen, Shenzhen, Guangdong 518055, China

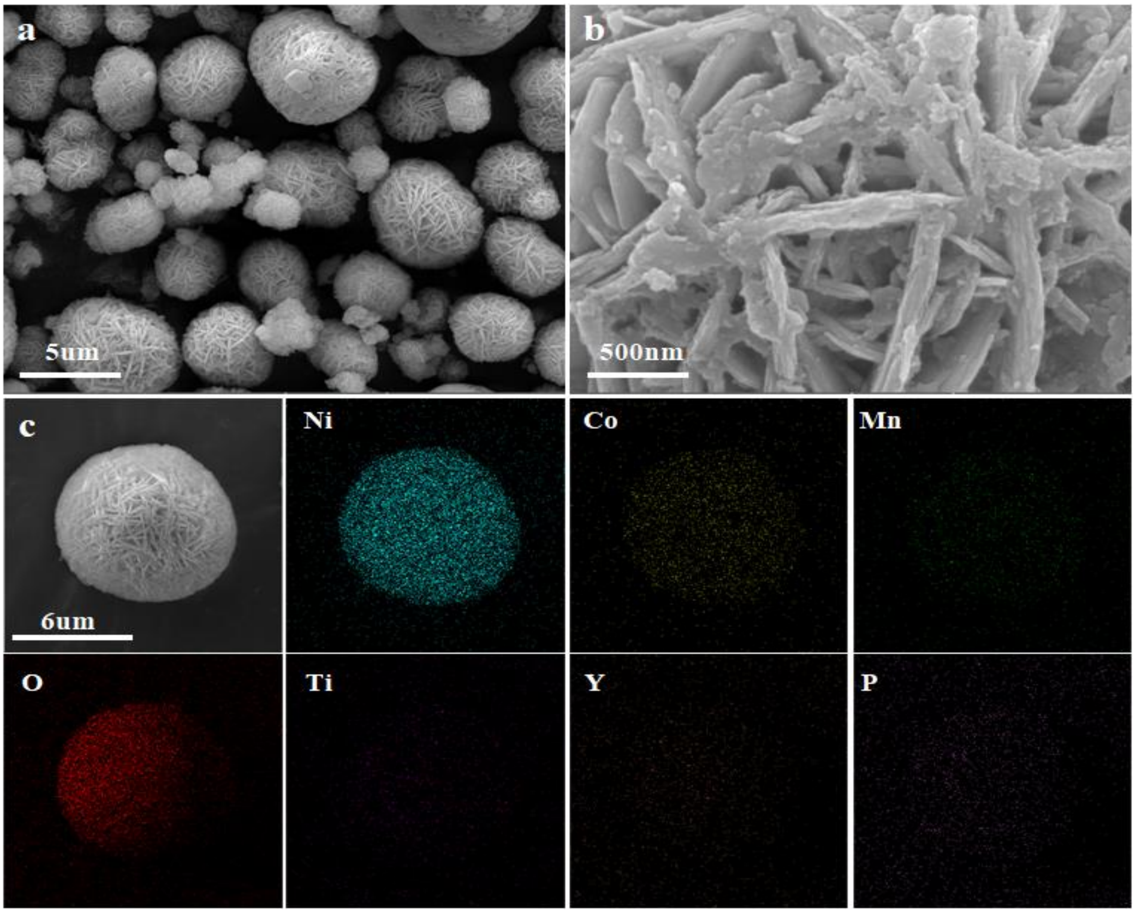

Supplementary Figure 1. (a, b) SEM images and (c) EDS elemental mapping of Ni, Co, Mn, O, Ti, Y and P for the 1% LYTP@ $Ni_{0.88}Co_{0.09}Mn_{0.03}(OH)_2$ precursor.

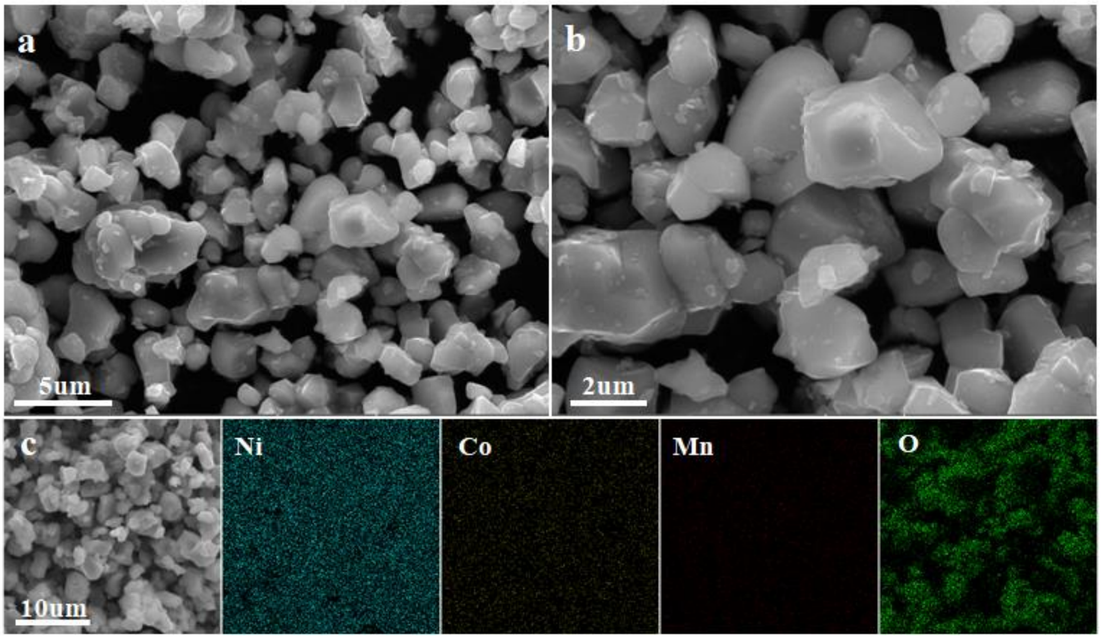

Supplementary Figure 2. (a, b) SEM images and (c) EDS elemental mapping of Ni, Co, Mn, O of the SC-NCM88 cathode.

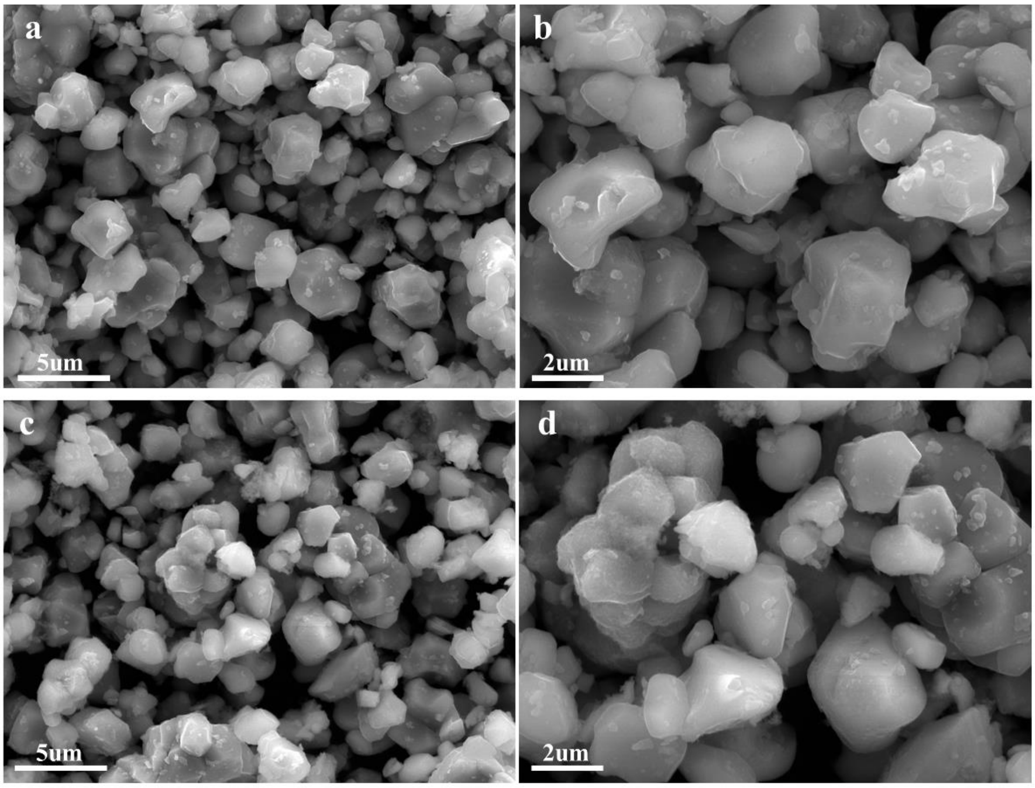

Supplementary Figure 3. SEM images of (a, b) 0.5% LYTP@SC-NCM88 and (c, d) 1% LYTP@SC-NCM88 cathode materials.

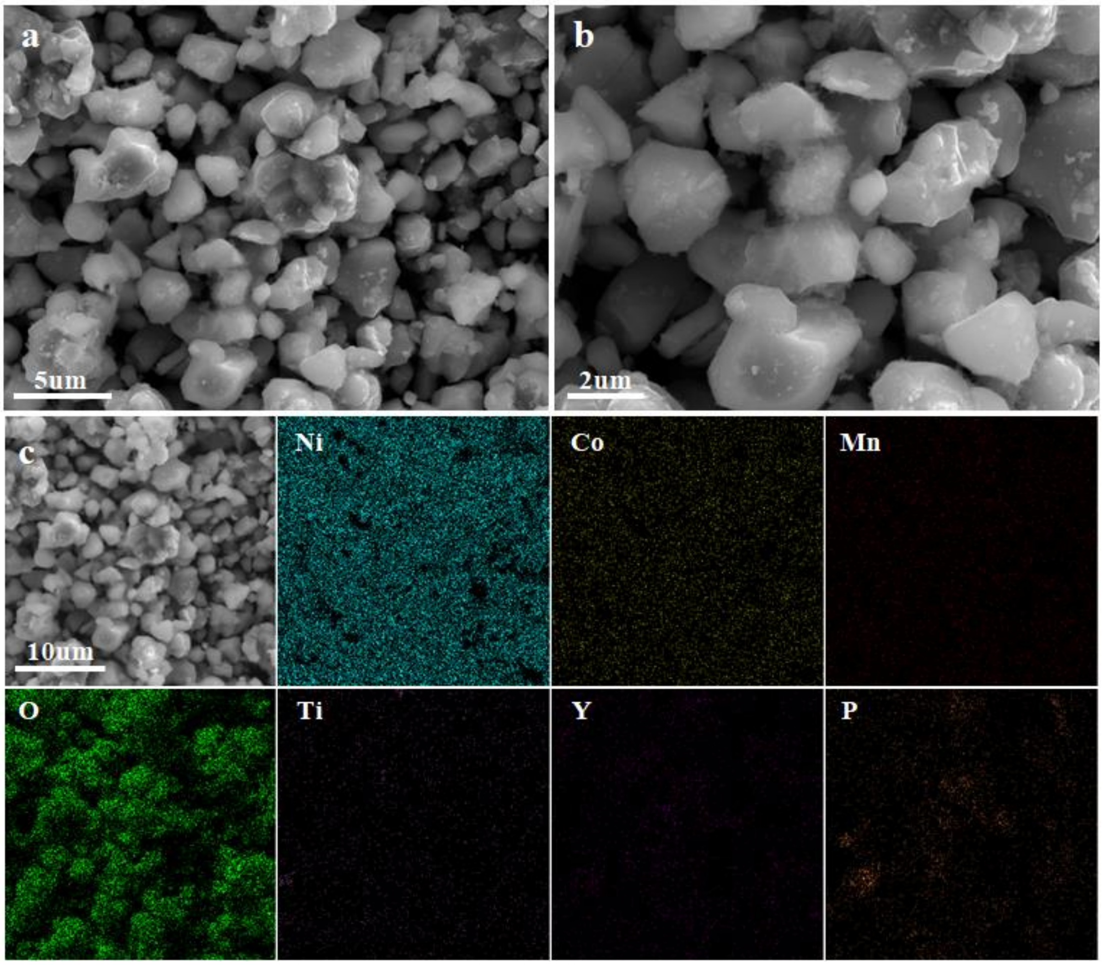

Supplementary Figure 4. (a, b) SEM images and (c) EDS elemental mapping of Ni, Co, Mn, O, Ti, Y, P for 3%LYTP@SC-NCM88 cathode material.

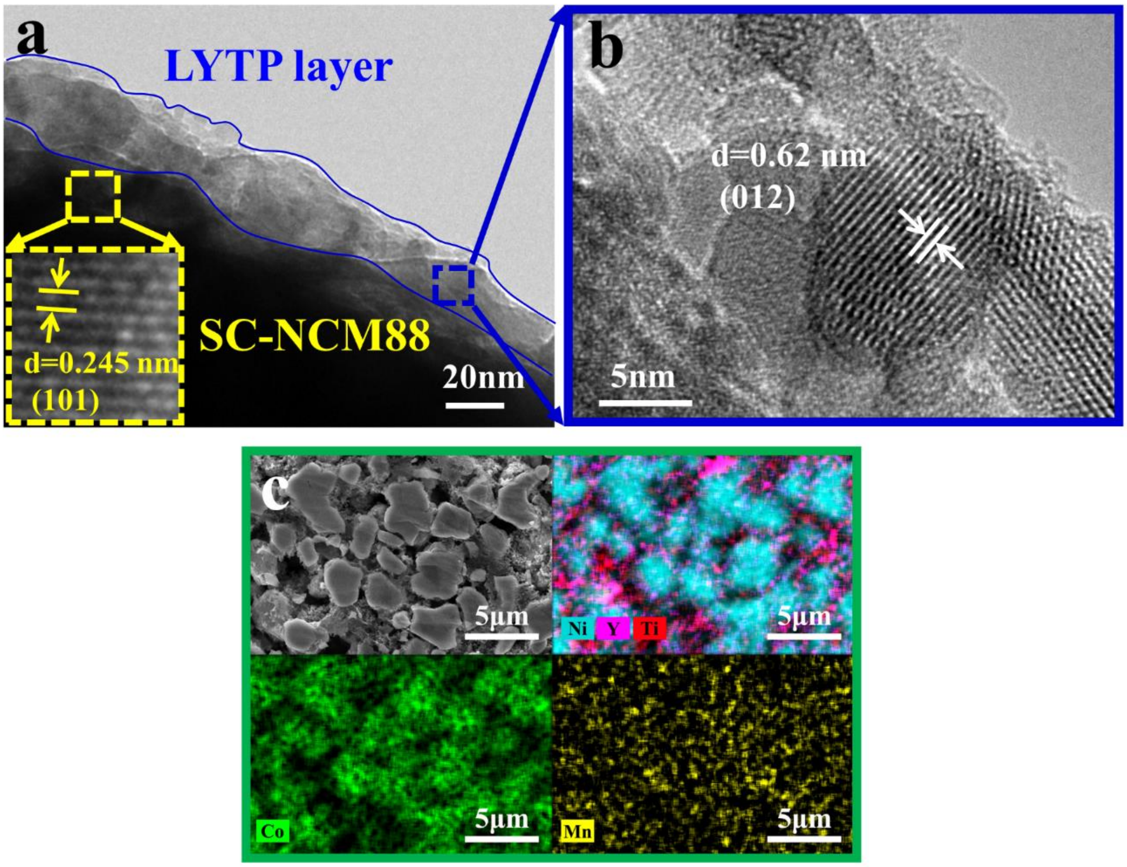

Supplementary Figure 5. (a) TEM, (b) HRTEM and (c) elemental SEM mappings of Ni, Co, Mn, Y, and Ti images of 1% LYTP@SC-NCM88.

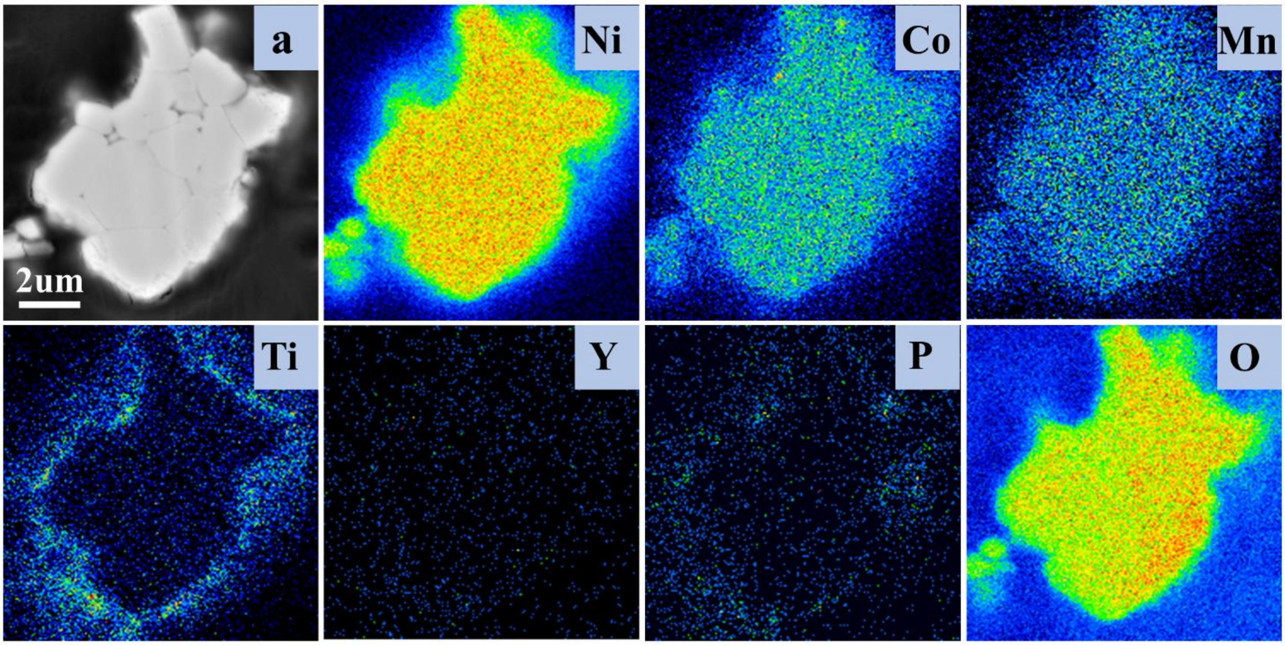

Supplementary Figure 6. The cross-sectional EPMA image of 1% LYTP@SC-NCM88 with the corresponding selected area LYTP mapping results of Ni, Co, Mn, Ti, and Y elements for double check.

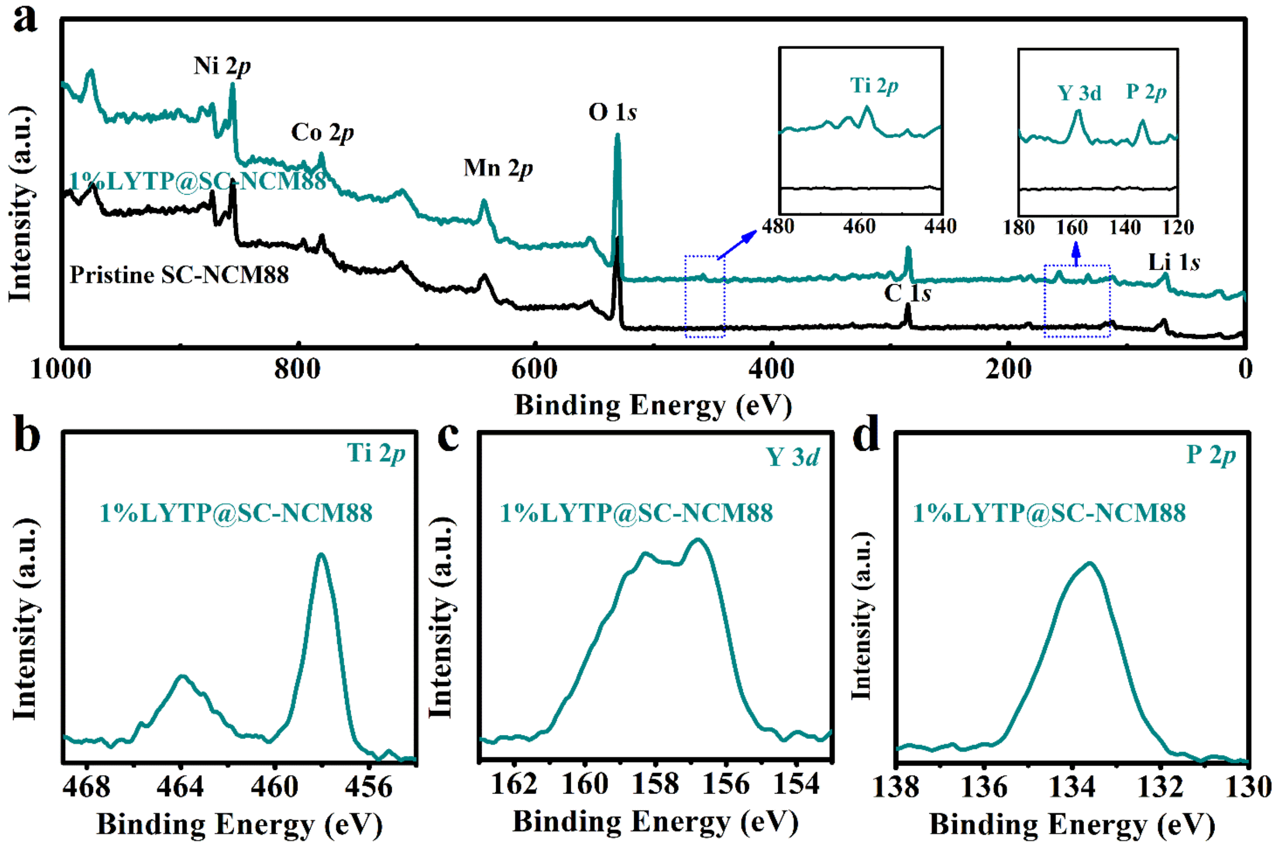

Supplementary Figure 7. (a) XPS full spectra of pristine SC-NCM88 and 1% LYTP@SC-NCM88. XPS spectra of 1% LYTP@ SC-NCM88 for (b) Ti 2*p*, (c) Y 3*d*, and (d) P 2*p*.

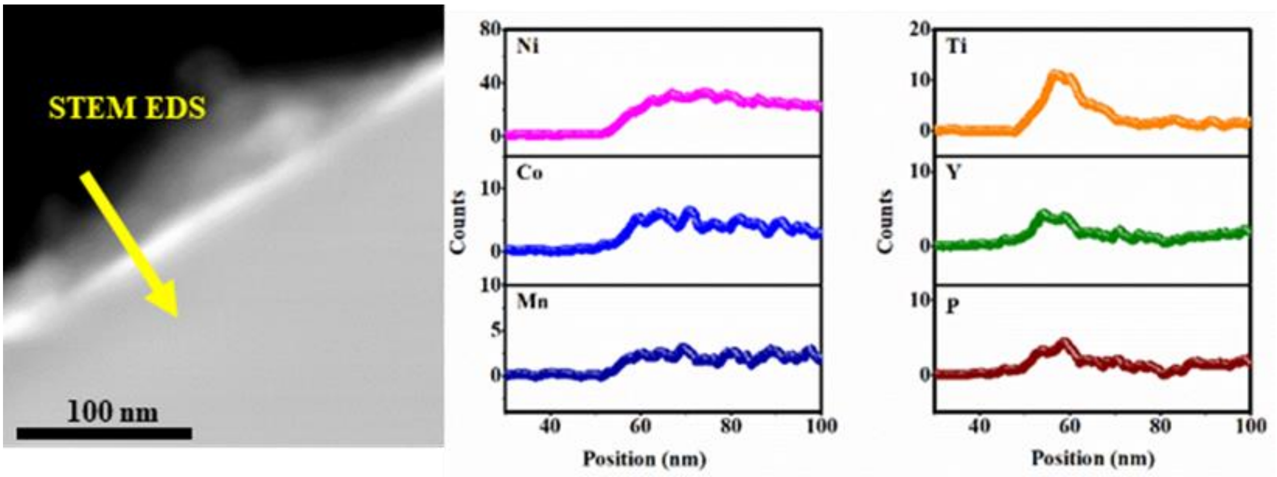

Supplementary Figure 8. TEM images and EDS elemental mapping of Ni, Co, Mn, O, Ti, Y, P for 1%LYTP@SC-NCM88 cathode material.

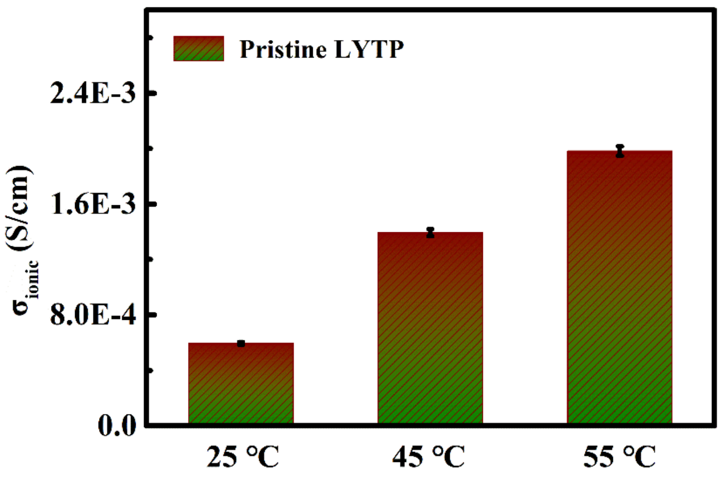

Supplementary Figure 9. Li-ion conductivity for pure $Li_{1.4}Y_{0.4}Ti_{1.6}(PO_4)_3$ at different temperatures. Error bars represent the standard deviations of three measurements for conductivity.

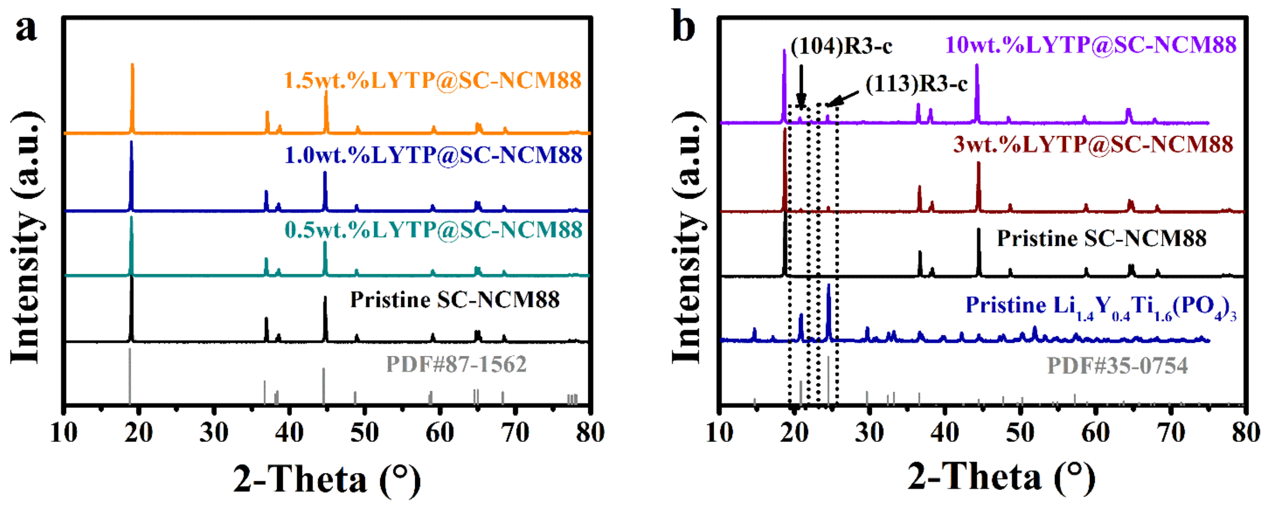

Supplementary Figure 10. Powder X-ray diffraction patterns for (a) pristine SC-NCM88, 0.5%LYTP@ SC-NCM88, 1% LYTP@ SC-NCM88, 1.5%LYTP@ SC-NCM88, (b) 3%LYTP@ SC-NCM88, 10%LYTP@ SC-NCM88 and pure $Li_{1.4}Y_{0.4}Ti_{1.6}(PO_4)_3$.

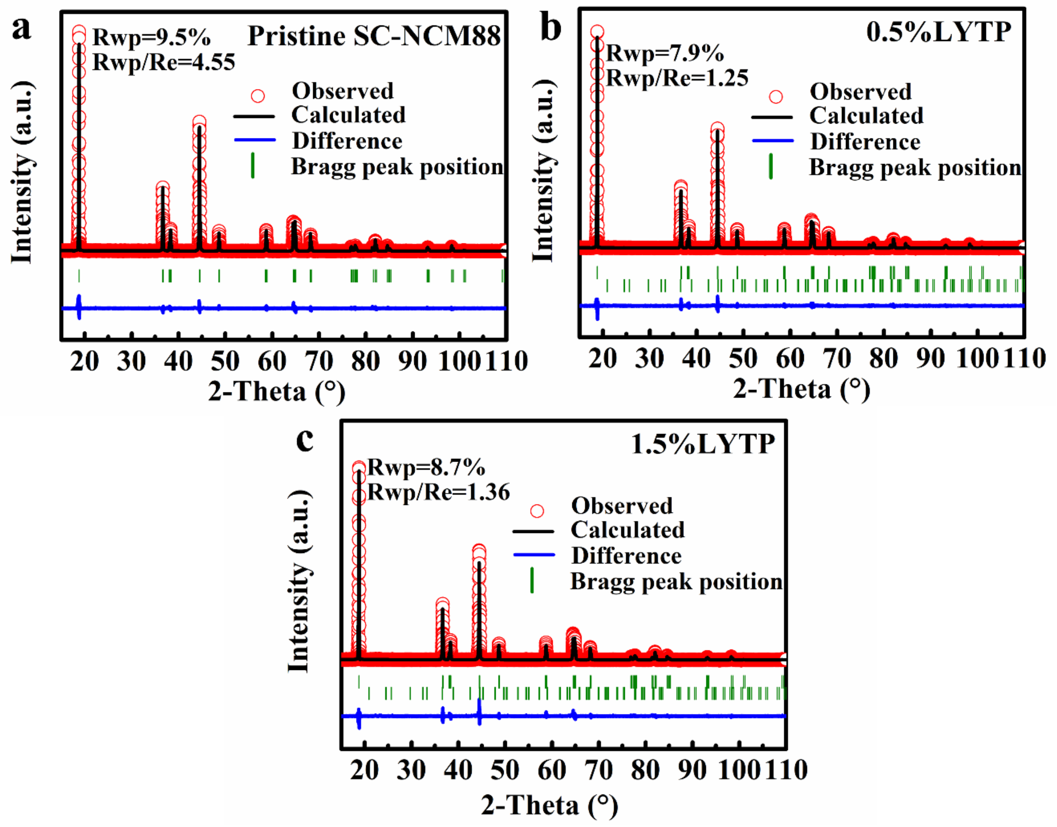

Supplementary Figure 11. Rietveld refinements of X-ray diffraction pattern for (a) pristine SC-NCM88, (b) 0.5 % LYTP and (c) 1.5 % LYTP.

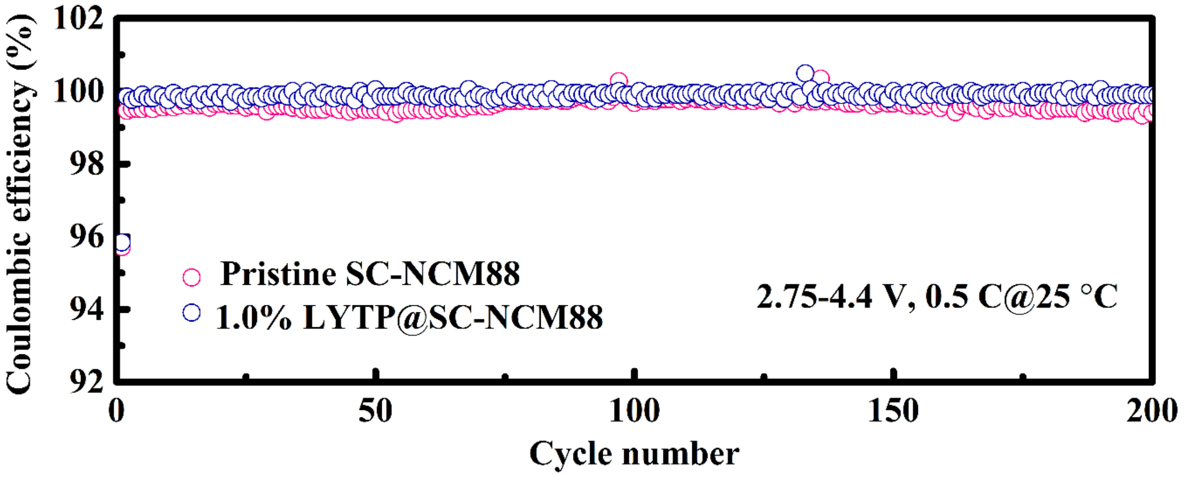

Supplementary Figure 12. Electrochemical evaluation for coin-type half cells. The Coulombic efficiency of pristine SC-NCM88 and 1% LYTP@SC-NCM88 against a lithium metal anode tested at 0.5 C under testing temperature of 25 °C.

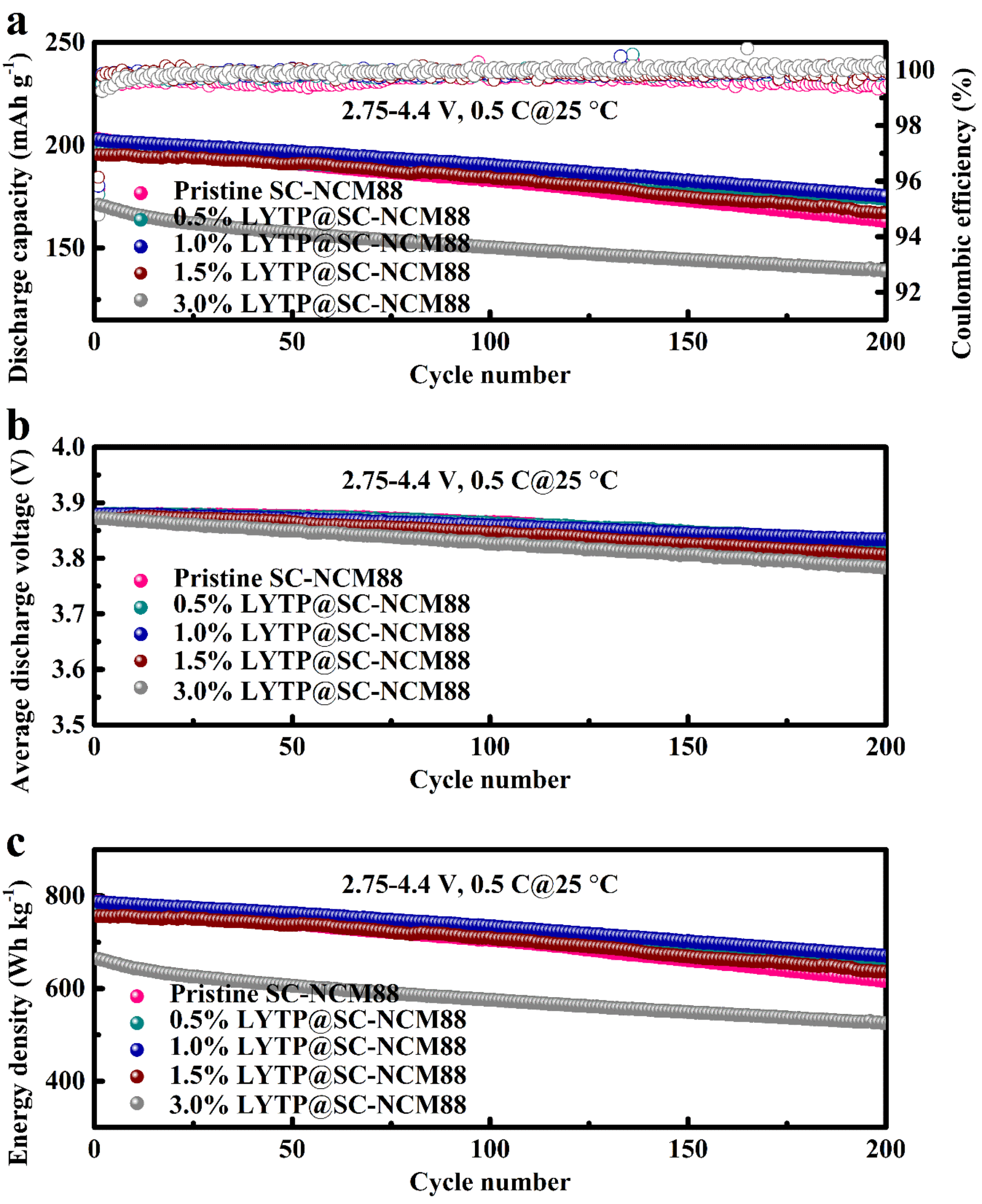

Supplementary Figure 13. (a) The discharge capacity, (b) discharging mid-point voltage, and (c) energy density for pristine SC-NCM88, 0.5%LYTP@SC-NCM88, 1%LYTP@SC-NCM88, 1.5%LYTP@SC-NCM88 and 3%LYTP@SC-NCM88 samples at 0.5 C with the voltage range of 2.75-4.4 V at temperature of 25°C.

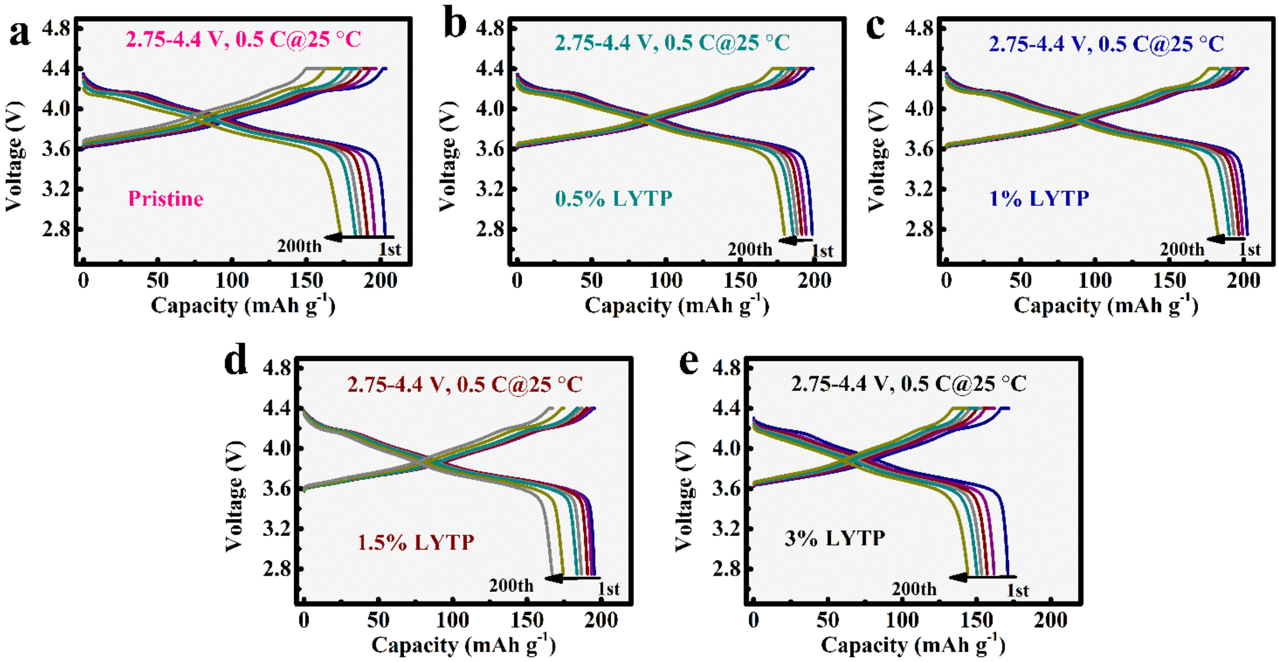

Supplementary Figure 14. Charge/discharge curves for (a) pristine SC-NCM88, (b) 0.5%LYTP@SC-NCM88, (c) 1% LYTP@SC-NCM88, (d) 1.5% LYTP@SC-NCM88 and (e) 3%LYTP@SC-NCM88 under different cycles at 0.5C with the voltage range of 2.75-4.4 V and temperature of 25°C.

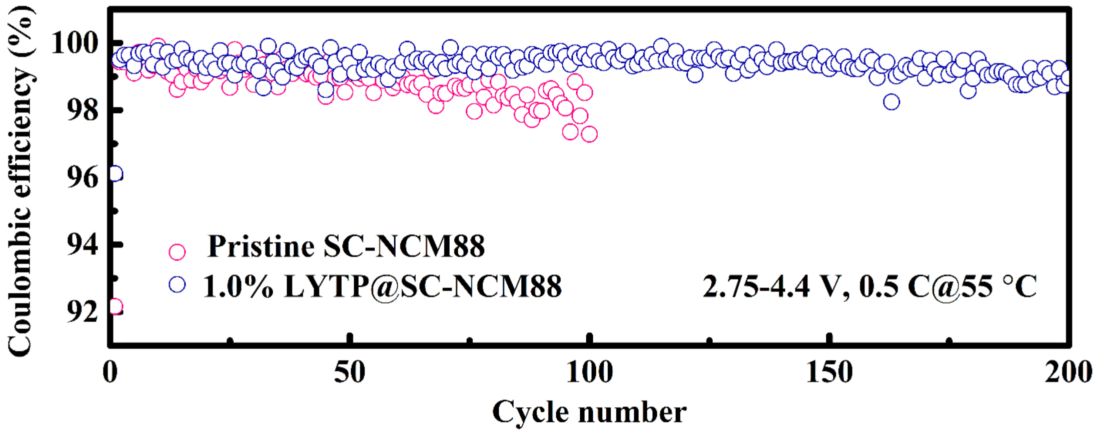

Supplementary Figure 15. Electrochemical evaluation for coin-type half cells. The Coulombic efficiency of pristine SC-NCM88 and 1% LYTP@SC-NCM88 against a lithium metal anode tested at 0.5 C under testing temperature of 55 °C.

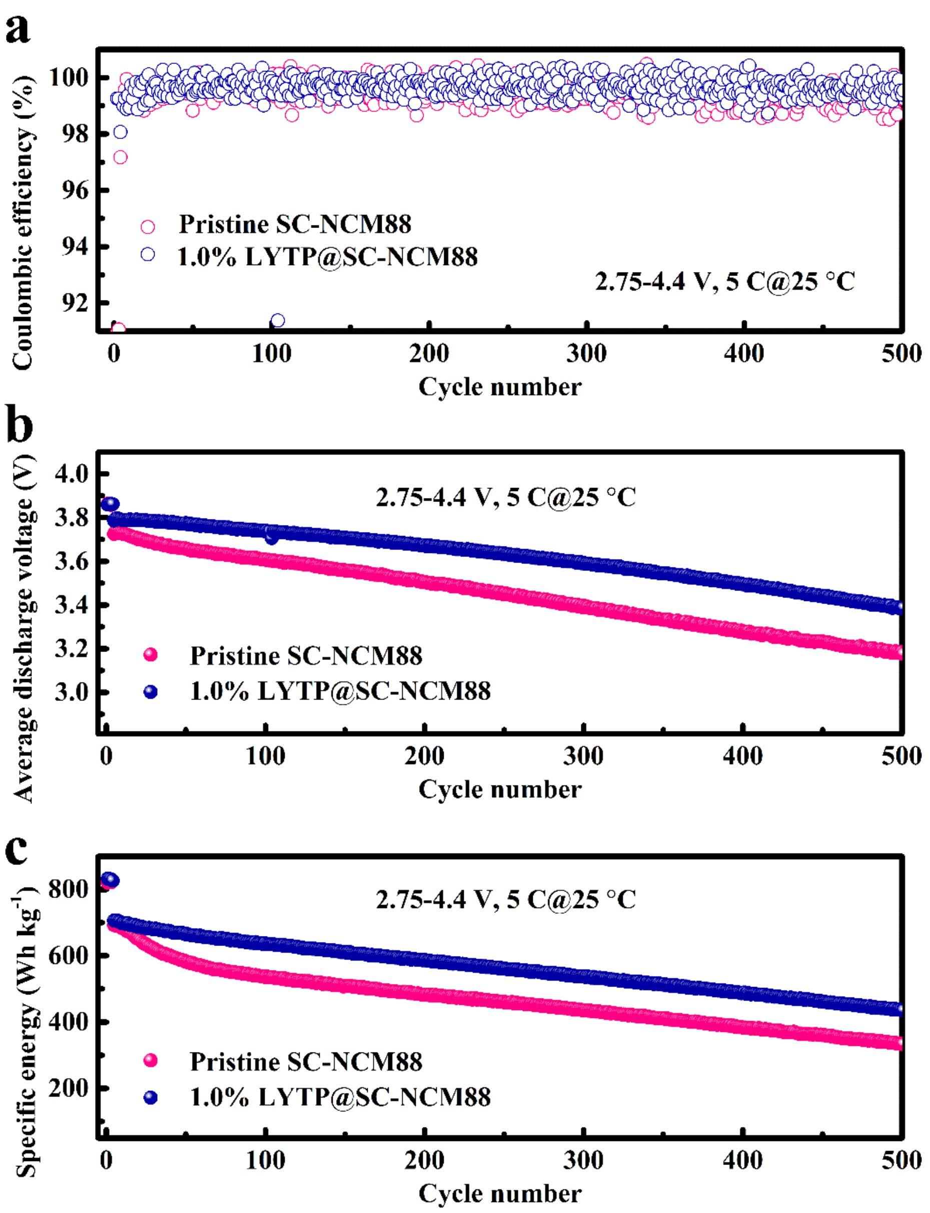

Supplementary Figure 16. The Coulombic efficiency (a), discharge middle voltage (b) and specific energy (c) for pristine and 1% LYTP@SC-NCM88 at different cycles at 5 C with the voltage range of 2.75-4.4 V and temperature of 25°C.

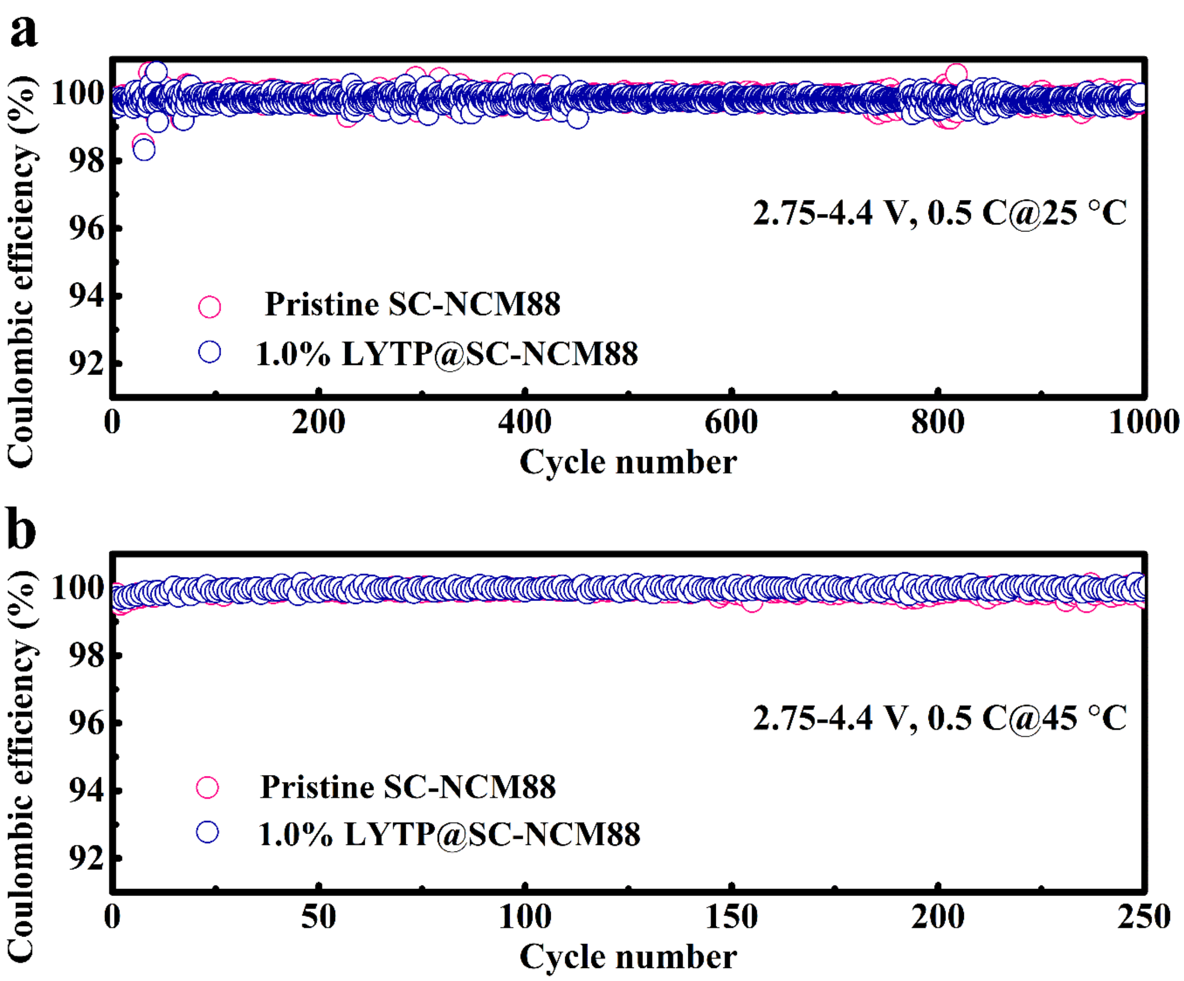

Supplementary Figure 17. Electrochemical evaluation for pouch-type full cells. Coulombic efficiency of pristine SC-NCM88 and 1% LYTP@SC-NCM88 against graphite anode tested at 0.5 C under testing temperature of (a) 25 ºC and (b) 45 ºC. All cells were cycled in the voltage range 2.75-4.4 V.

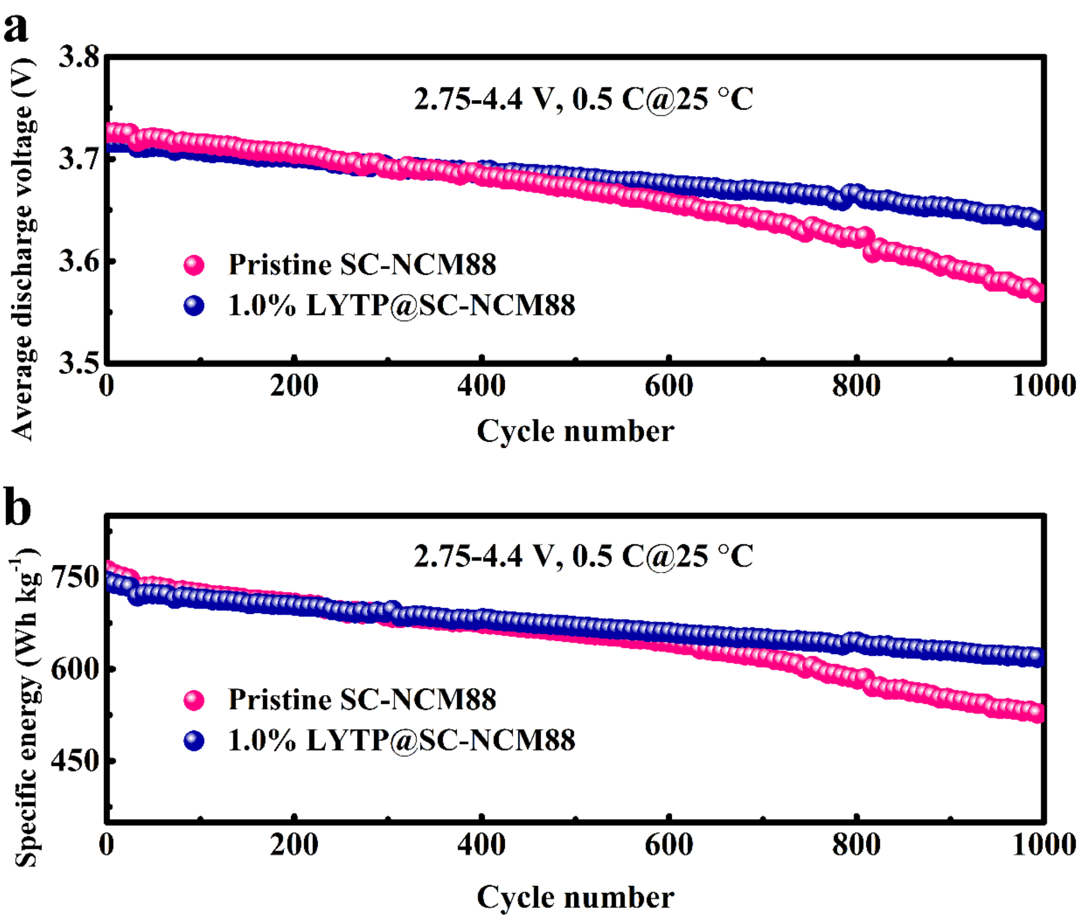

Supplementary Figure 18. (a) The discharge middle voltage and (b) specific energy for pristine SC-NCM88 and 1% LYTP@SC-NCM88 at different cycles with the voltage range of 2.75-4.4 V and temperature of 25°C for pouch-type full cells.

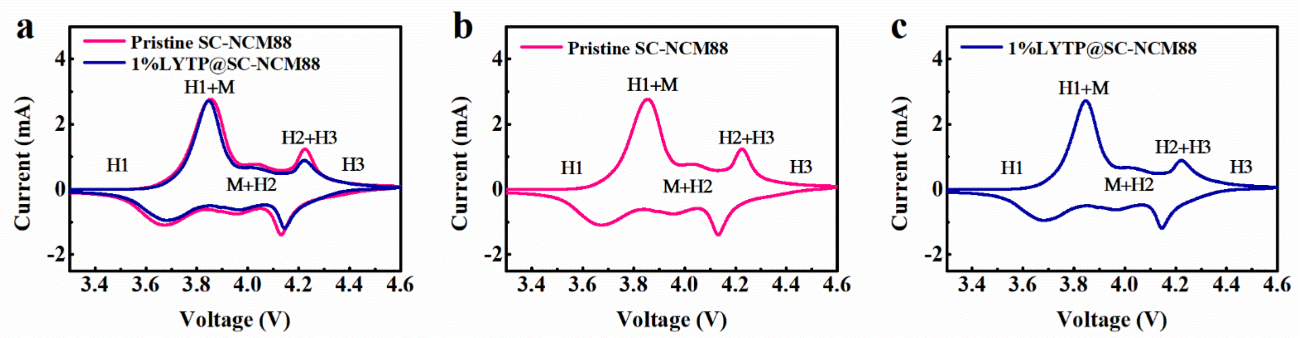

Supplementary Figure 19. The overlapped and individual CV curves for (a, b) pristine SC-NCM88 and (a, c) 1% LYTP@SC-NCM88.

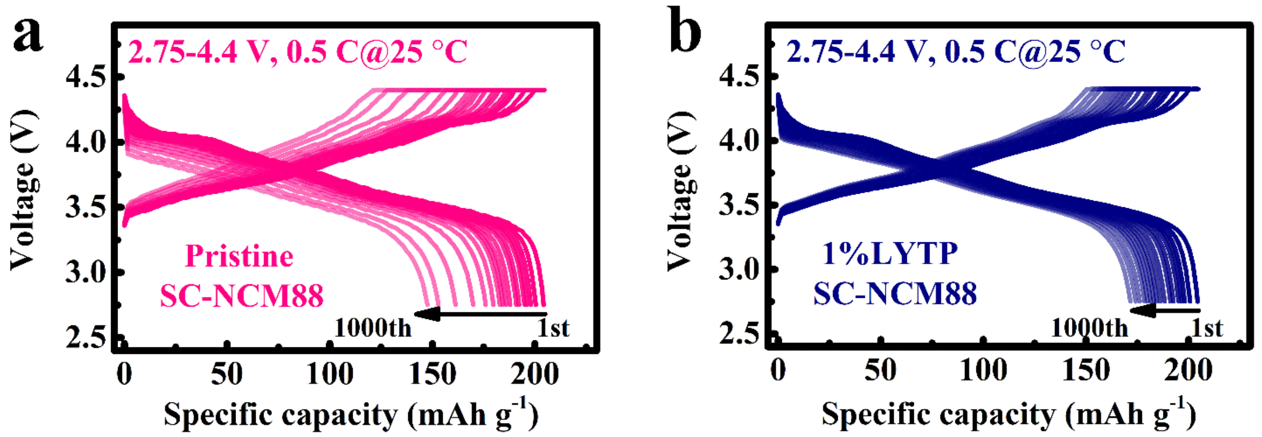

Supplementary Figure 20. Charge/discharge curves for (a) pristine SC-NCM88 and (b) 1% LYTP@SC-NCM88 at different cycles with the voltage range of 2.75-4.4 V and temperature of 25°C for pouch-type full cells.

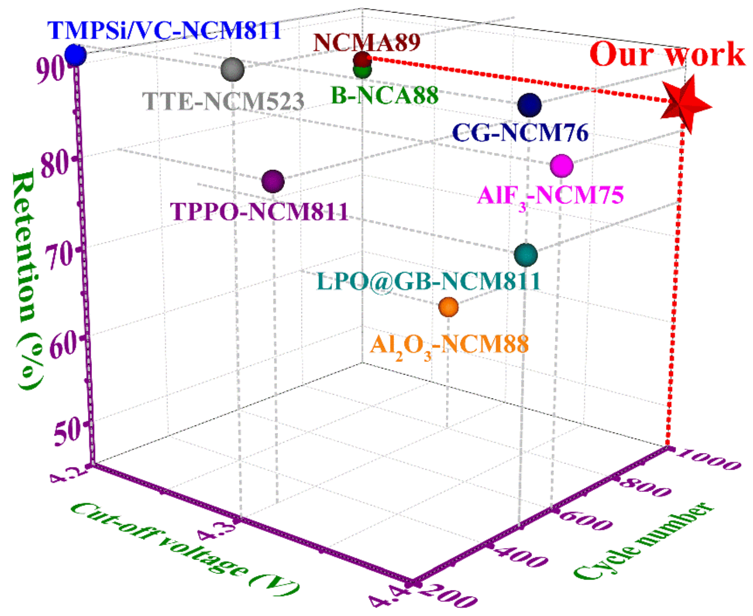

Supplementary Figure 21. Comparison of cycling performance of the 1% LYTP@SC-NCM88 pouch cell and previously reported pouch cells with Ni-rich NCM/NCA cathodes.

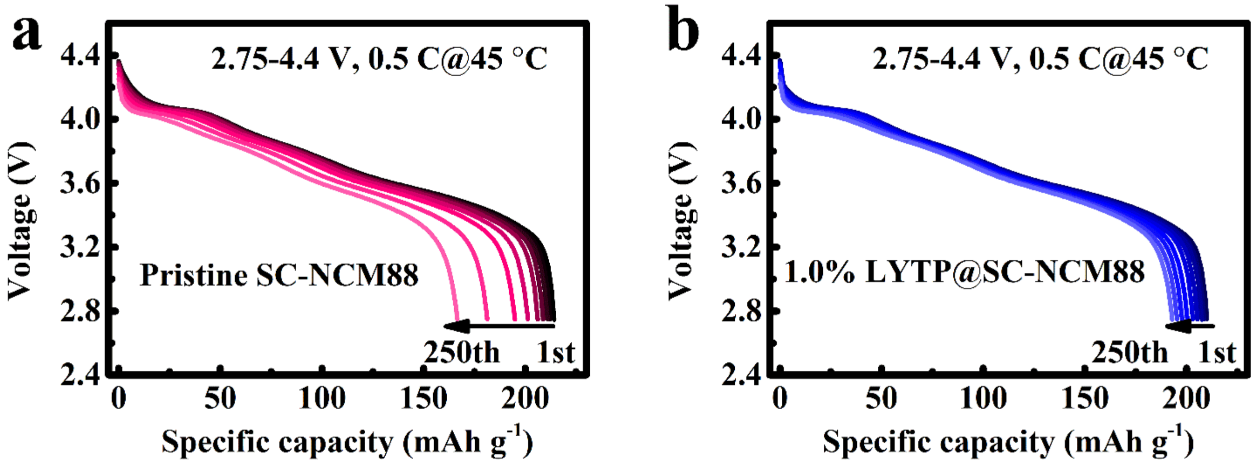

Supplementary Figure 22. Discharge curves for (a) pristine SC-NCM88 and (b) 1% LYTP@ SC-NCM88 at different cycles in the voltage range of 2.75-4.4 V and temperature of 45°C for pouch-type full cells.

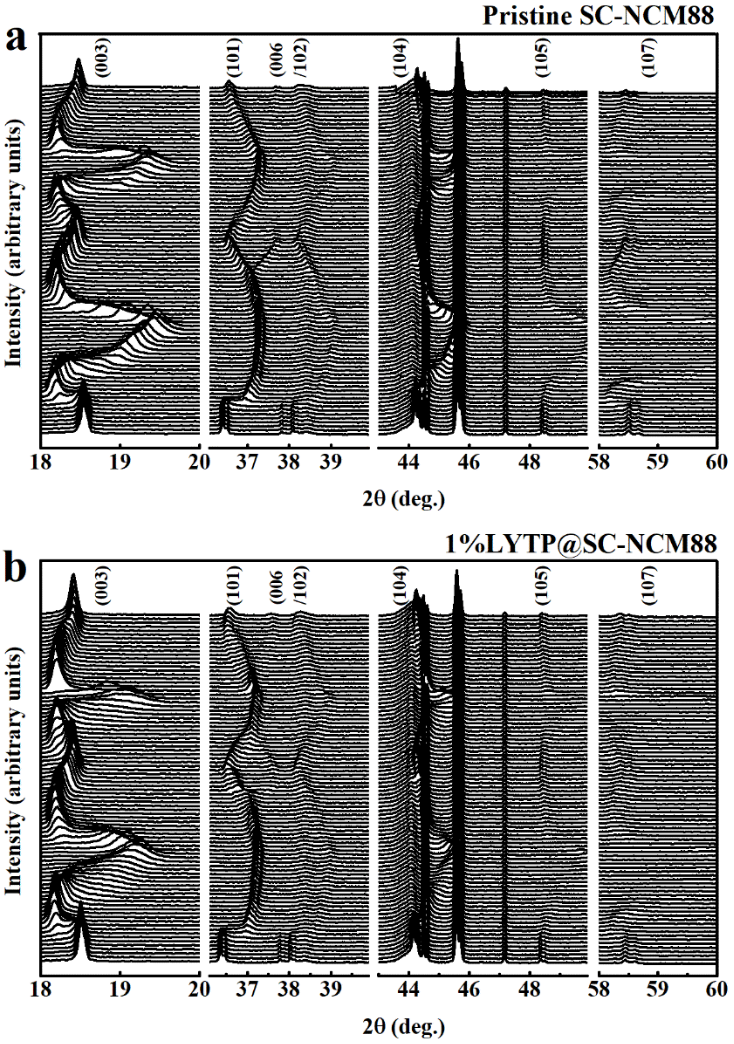

Supplementary Figure 23. In situ XRD stacked patterns for (a) pristine SC-NCM88 and (b) 1% LYTP@SC-NCM88 cathodes during the first 2 cycle charge-discharge process.

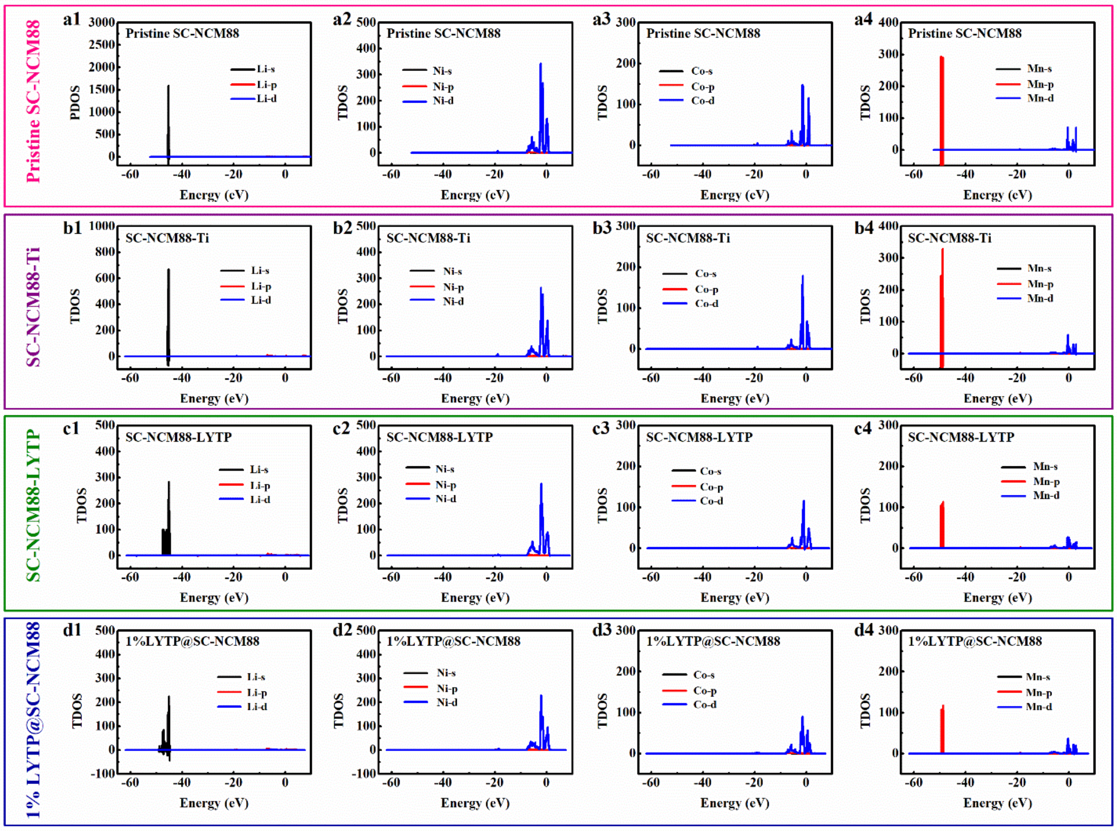

Supplementary Figure 24. The partial density of states (PDOS) of Li, Ni, Co and Mn elements for (a) pristine SC-NCM88, (b) surface Ti-atoms doping (SC-NCM88-Ti), (c) LYTP nanolayer coating (SC-NCM88-LYTP) and (d) NCM with surface doping and LYTP coating (1% LYTP@SC-NCM88).

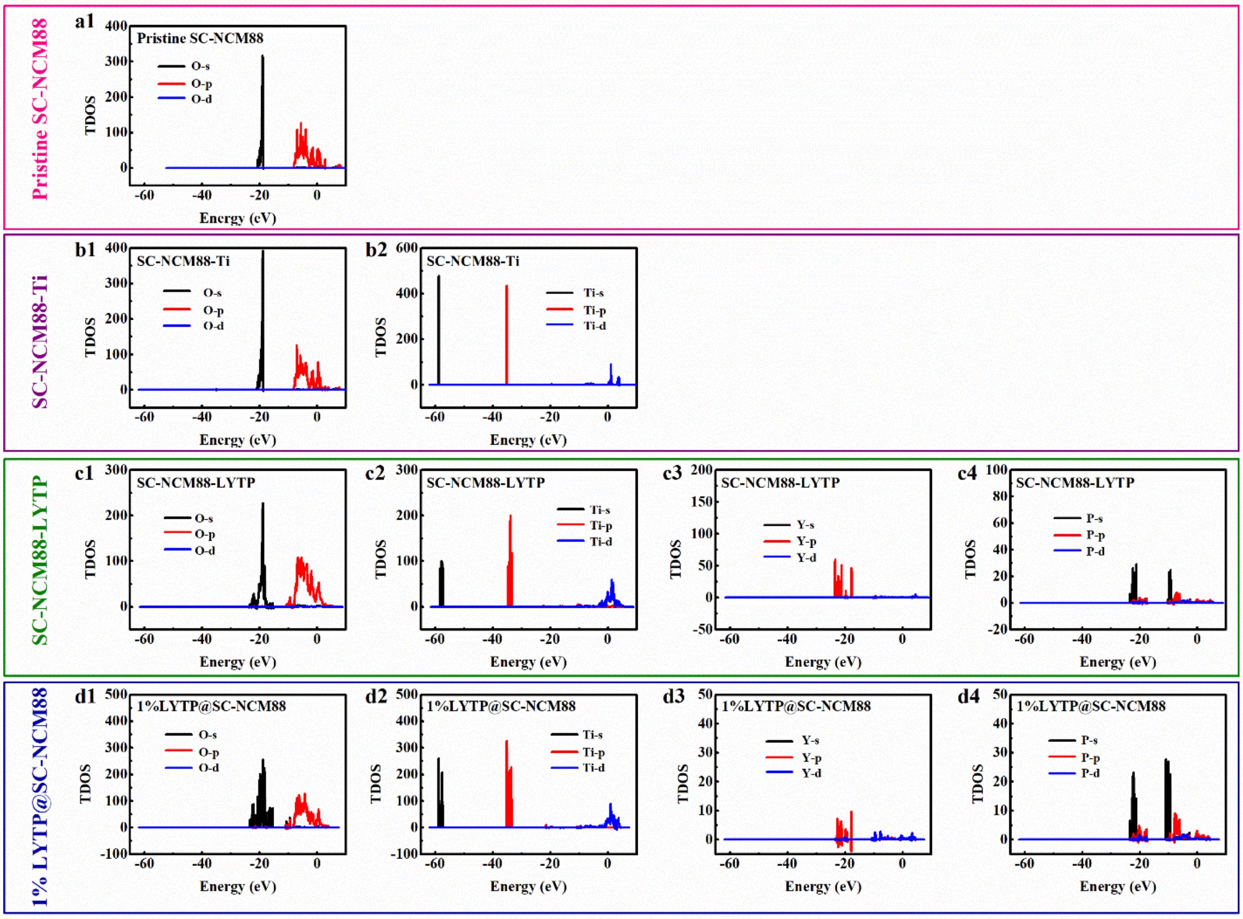

Supplementary Figure 25. The partial density of states (PDOS) of O, Ti, Y and P elements for (a) pristine SC-NCM88, (b) surface Ti-atoms doping (SC-NCM88-Ti), (c) LYTP nanolayer coating (SC-NCM88-LYTP) and (d) NCM with surface doping and LYTP coating (1% LYTP@SC-NCM88).

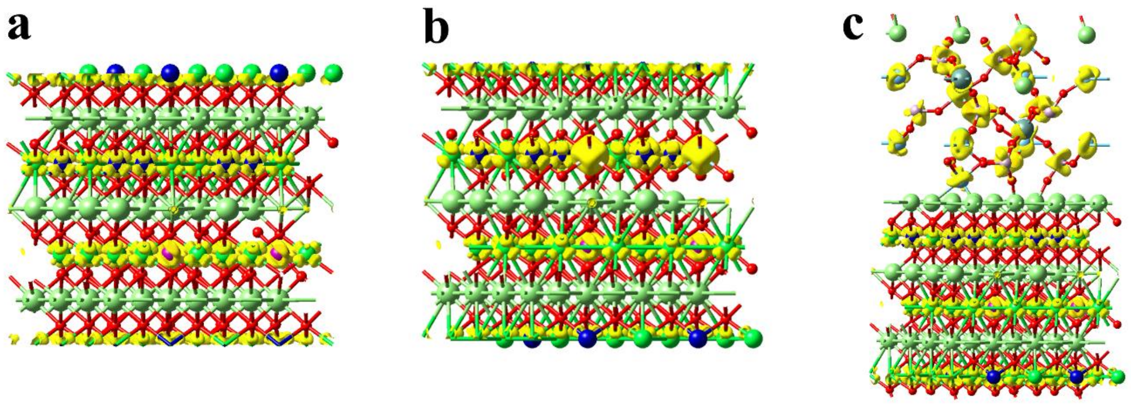

Supplementary Figure 26. Relaxed crystal structures for (a) pristine SC-NCM88, (b) surface Ti-atoms doping (SC-NCM88-Ti), and (c) LYTP nanolayer coating (SC-NCM88-LYTP).

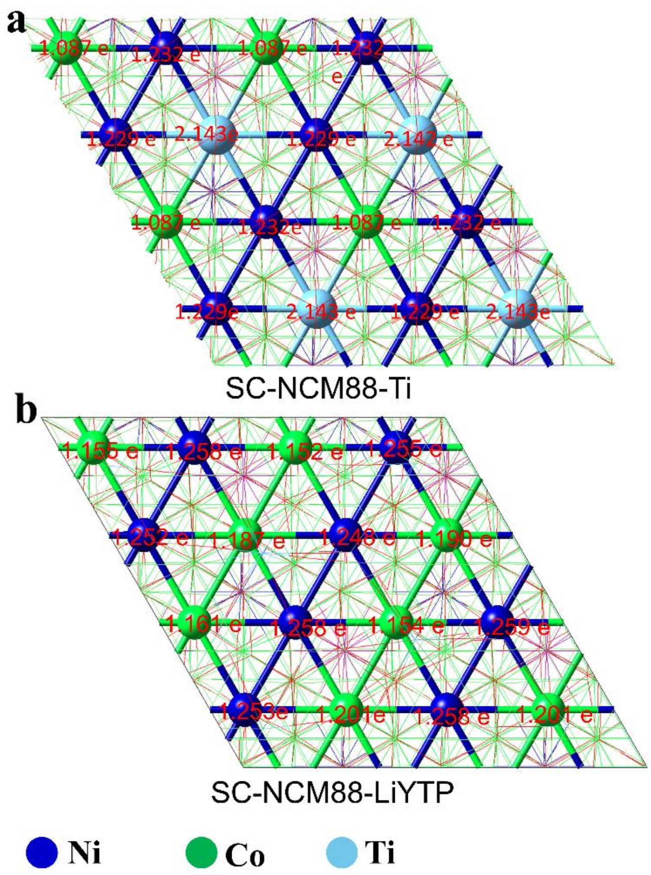

Supplementary Figure 27. The Bader charge transfer for (a) surface Ti-atoms doping (SC-NCM88-Ti) and (b) LYTP nanolayer coating (SC-NCM88-LYTP).

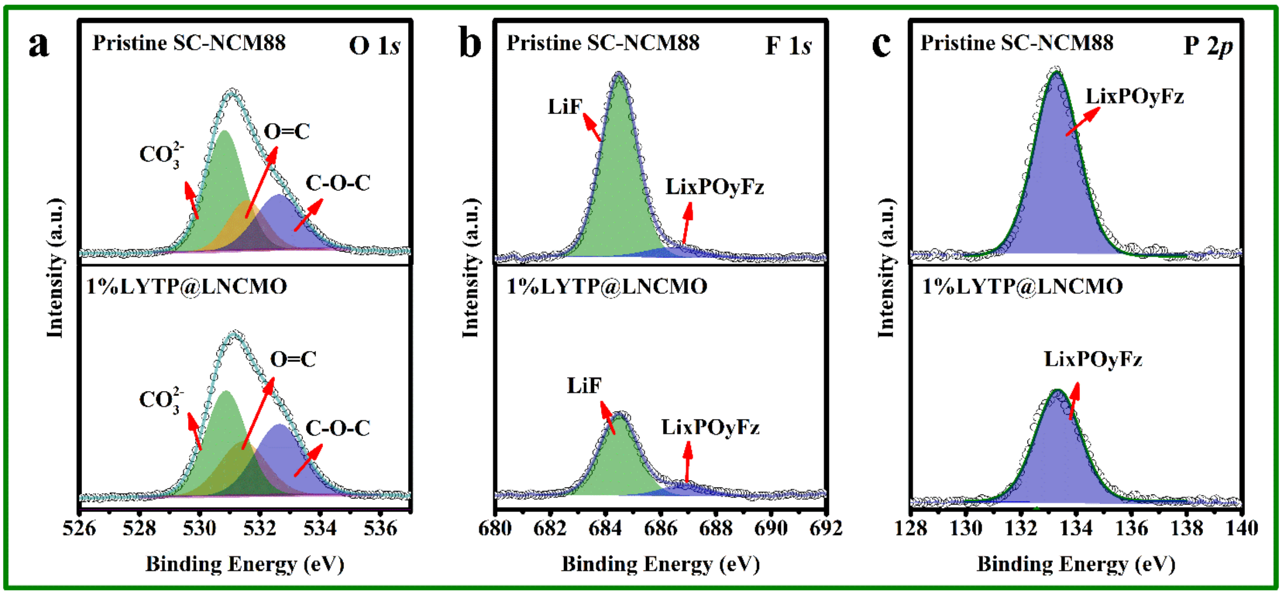

Supplementary Figure 28. The XPS spectra of (a) O 1*s*, (b) F 1*s* and (c) P 2*p* for pristine SC-NCM88 and 1% LYTP@SC-NCM88 harvested from pouch-type full cells with a cut-off voltage of 4.4 V after 200 cycles.

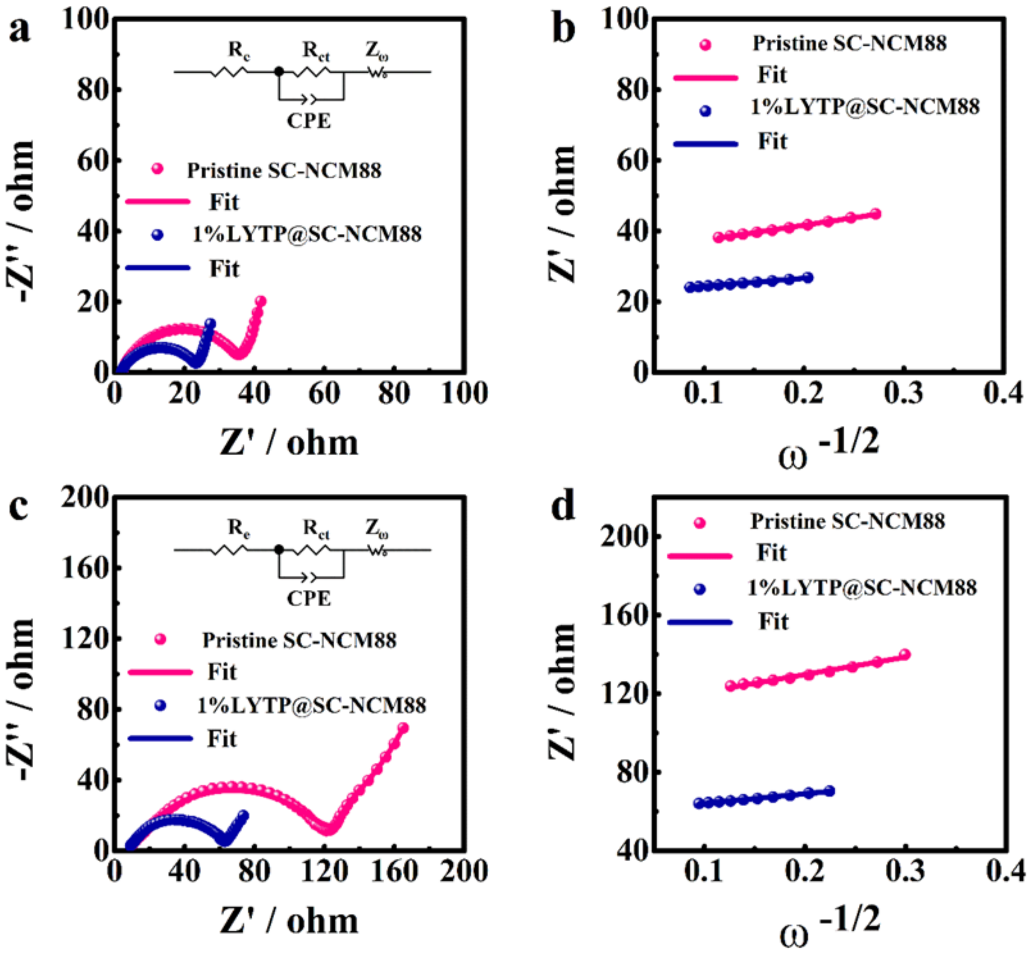

Supplementary Figure 29. Nyquist plots of pristine SC-NCM88 and 1% LYTP@ SC-NCM88 cathodes recorded (a) before cycling and (c) after 200 cycles. Z' *vs.* $\omega^{-1/2}$ plots in the low frequency region obtained from the corresponding Nyquist plots (b) before and (d) after cycling.

The equivalent circuit modelling is applied to fit the EIS spectra, which includes three regions:

(1) The electrolyte resistance (R_e).

(2) The charge transfer resistance (R_{ct})

(3) The Warburg impedance (Z_ω) representing Li^+ diffusion.

(4) Constant phase angle element (CPE), which is compensatory to fit the EIS spectra.

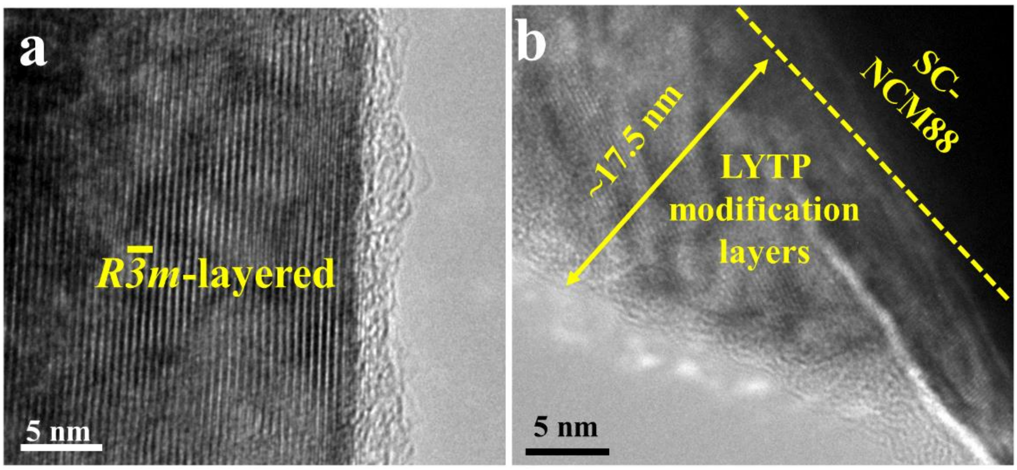

Supplementary Figure 30. (a, b) HRTEM images of various surface regions for 1% LYTP@ SC-NCM88 after 200 cycles.

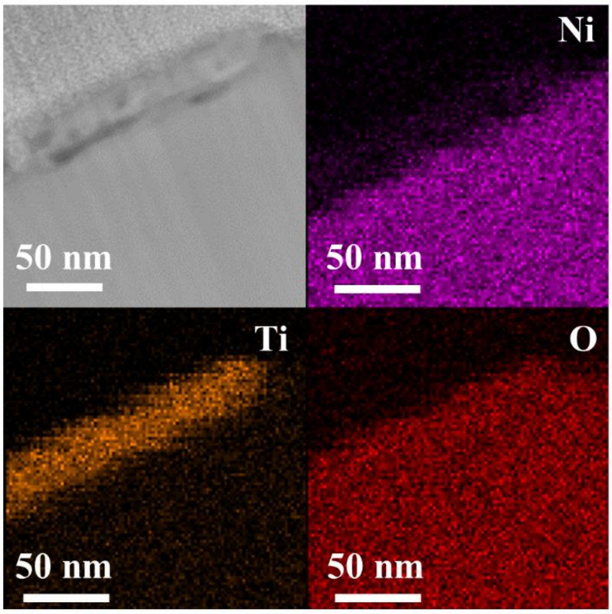

Supplementary Figure 31. STEM elemental mappings of Ni, Ti, and O for 1% LYTP@ SC-NCM88 after 200 cycles.

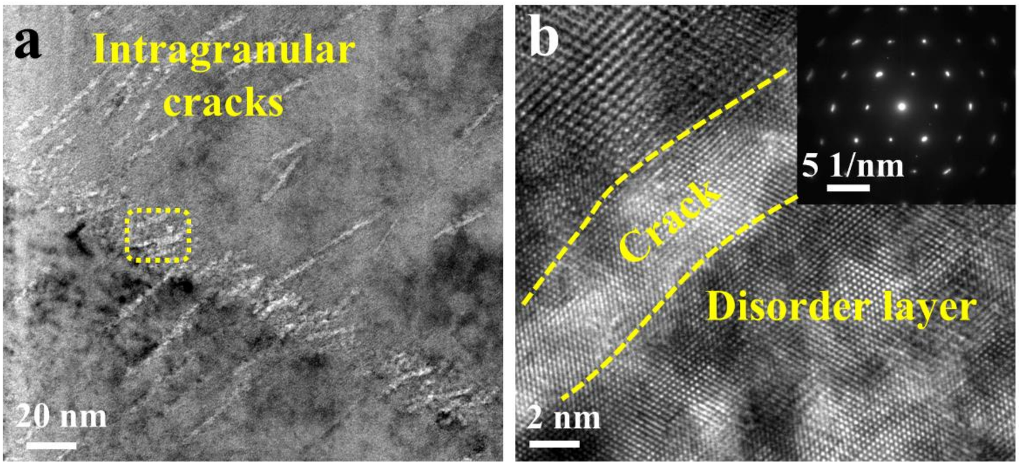

Supplementary Figure 32. (a) TEM and (b) HRTEM images of pristine SC-NCM88.

Supplementary Table 1. Chemical compositions of the pristine SC-NCM88 and modified LYTP@SC-NCM88 cathodes measured by inductively coupled plasma analysis.

Sample	ICP (wt%)					
	Ni	Co	Mn	Ti	Y	P
Pristine SC-NCM88	52.831	5.498	1.802	/	/	/
0.5%LYTP@SC-NCM88	52.714	5.527	1.568	0.092	0.043	0.112
1.0%LYTP@SC-NCM88	52.271	5.260	1.839	0.196	0.092	0.238
1.5%LYTP@SC-NCM88	51.770	5.591	1.608	0.282	0.129	0.339
Sample	Measured molar ration					
	Ni	Co	Mn	Ti	Y	P
Pristine SC-NCM88	0.877	0.091	0.032	/	/	/
0.5%LYTP@SC-NCM88	0.881	0.092	0.028	0.0019212	0.0004879	0.0036238
1.0%LYTP@SC-NCM88	0.878	0.088	0.033	0.0040852	0.0010304	0.007699
1.5%LYTP@SC-NCM88	0.874	0.094	0.029	0.0058903	0.0014535	0.0109607

Supplementary Table 2. The ionic conductivities of pristine LYTP, pristine SC-NCM88, and 1% LYTP@SC-NCM88.

Sample	Ionic conductivity (S/cm)		
	25°C	45°C	55°C
Pristine LYTP	5.939(4)E-04	1.394(3)E-04	1.983(2)E-04
Pristine SC-NCM88	6.323(2)E-05	1.379(4)E-04	1.536(8)E-04
1.0%LYTP@SC-NCM88	9.497(5)E-05	1.560(3)E-04	1.759(3)E-04

Supplementary Table 3. The Electron conductivities of pristine SC-NCM88 and 1% LYTP@SC-NCM88.

Sample	Electron conductivity (S/cm)
Pristine SC-NCM88	1.419(3)E-02
1.0%LYTP@SC-NCM88	2.083(4)E-02

Supplementary Table 4. Comparison of the cycle stability of 1% LYTP@LNCMO with the Ni-rich based materials as reported elsewhere, as the cathode materials for pouch full cells.

Sample	Charge/Discharge rate	Voltage range	1st capacity (mAh g^{-1})	End capacity (mAh g^{-1}/)	Capacity retention /cycles	Type of cell configuration/ anode material	Electrolyte used for testing	References
**1%LYTP@NCM88**	0.5C/0.5C	2.75-4.4V	200.1	170	85%/1000	Pouch cell/graphite	1.1M $LiPF_6$ EC/EMC/DEC(3:5:2 vol%)+1wt%VC	Our work
**LPO@GB-NCM811**	1C/1C	2.7-4.4V	/	/	74%/500	Coin cell/graphite	/	1
**AlF_3 coated NCM751515**	1C/1C	2.5-4.4V	190	155.8	82%/600	Not mentioned/graphite	1.2M $LiPF_6$ EC/EMC(3:7 vol%)+2vol%VC	2
**CG-NCM761014**	0.3C/0.3C	2.5-4.4V	179.3	159.6	89%/500	Pouch cell/graphite	1.2M $LiPF_6$ EC/EMC(3:7 vol%)+2wt%VC	3
**Al_2O_3 modified NCM88**	0.5C/1C	3.0-4.3V	198.1	118.7	59.95%/800	Coin cell/Li foil	1.0M $LiPF_6$ EC/DMC(3:7 vol%)+0.01gAl_2O_3	4
**TPPO in NCM811**	0.5C/0.5C	2.8-4.3V	187	149.6	80%/295	Swagelok cell/graphite	1.0M $LiPF_6$ EC/EMC(3:7 wt%)+0.5wt%TPPO	5
**3 wt % TTE in NCM523**	0.5C/0.5C	2.8-4.3V	155.5	143.2	92.1%/200	Coin cell/graphite	1.0M $LiPF_6$ EC/EMC(1:1 wt%)+3wt% TTE	6
**B-doping NCA88**	1C/1C	2.75-4.2V	188	156.8	83.4%/1000	Pouch cell/graphite	1.2M $LiPF_6$ EC/EMC(3:7 vol%)+2wt%VC	7
**NCMA89**	1C/1C	2.75-4.2V	/	/	84.5%/1000	Pouch cell/MCMB	1.2M LiPF6 EC/EMC(3:7 vol%)+2wt%VC	8
**1% TMPSi NCM811**	0.3C/0.3C	2.75-4.2V	/	/	91%/200	Pouch cell/graphite	1.0M $LiPF_6$ EC/DMC(5:5 vol%)+1vol%TMPSi+1vol%VC	9
**$Li_{1.15}Mn_{0.55}Ni_{0.2}Co_{0.1}O_2$**	1C/1C	2.0-4.7V	226	196.8	87.1%400	Pouch cell/graphite	1.0M LiPF6 EC/DMC(1:1 vol%)	10
**$Li_{1.2}Ni_{0.27}Mn_{0.40}Co_{0.13}O_2$**	0.2C/0.2C	2.0-4.6V	190	185	94.9%/150	Coin cell/graphite	1.0M LiPF6 EC/DMC(1:1 vol%)	11
**Ni-doping $LiCoO_2$**	0.2C/0.2C	3.0-4.35 V	/	/	85%/500	Pouch cell/graphite	1.0M LiPF6 EC/DMC/DEC(1:2:1 vol%)	12
**$LiNi_{0.5}Mn_{1.5}O_4$**	0.2C/0.2C	3.5-4.9 V	112.9	113.3	100.4%/100	Coin cell/SiOx-C	1.0M LiPF6 EC/DEC/EMC(1:1:1 vol%)+1wt%FEC+1wt%PCS	13

Notes: All the cell tests have been carried out at 25 °C.

Supplementary Table 5. Fitting results for pristine SC-NCM88 and 1% LYTP@ SC-NCM88 samples obtained from EIS results.

**Samples**	**R_e** (Ω)	**R_{ct}** (Ω)	**D_{Li}** ($cm^2\ s^{-1}$)	**CPE**
Pristine LNCMO-fresh	2.12(8)	32.1(4)	$2.4(5)\times10^{-14}$	$2.299(6)\ \times10^{-5}$
1%LYTP@LNCMO-fresh	1.97(6)	20.4(7)	$8.3(7)\times10^{-14}$	$3.840(6)\ \times10^{-5}$
Pristine LNCMO-cycled	8.72(6)	114.7(9)	$5.5(2)\times10^{-15}$	$2.897(2)\times10^{-5}$
1%LYTP@LNCMO-cycled	8.05(9)	54.2(9)	$1.8(6)\times10^{-14}$	$2.090(2)\times10^{-5}$

Supplementary References

1. Cheng X., Zheng J., Lu J., Li Y., Yan P., Zhang Y. Realizing superior cycling stability of Ni-Rich layered cathode by combination of grain boundary engineering and surface coating. *Nano Energy* **62**, 30-37 (2019).
2. Oh P., Oh S.-M., Li W., Myeong S., Cho J., Manthiram A. High-Performance heterostructured cathodes for lithium-ion batteries with a Ni-rich layered oxide core and a Li-rich layered oxide dhell. *Adv. Sci.* **3**, 1600184 (2016).
3. Liao J.Y., Oh S.M. & Manthiram A. Core/Double-Shell type gradient Ni-rich $LiNi_{0.76}Co_{0.10}Mn_{0.14}O_2$ with high capacity and long cycle life for lithium-ion batteries. *ACS Appl. Mater. Interfaces* **8**, 24543-24549 (2016).
4. Sun Y.-Y., Liu S., Hou Y.-K., Li G.-R., Gao X.-P. In-situ surface modification to stabilize Ni-rich layered oxide cathode with functional electrolyte. *J. Power Sources* **410-411**, 115-123 (2019).
5. Beltrop K. *et al.* Triphenylphosphine oxide as highly effective electrolyte additive for graphite/NMC811 lithium ion cells. *Chem. Mater.* **30**, 2726-2741 (2018).
6. Heng S. *et al.* Fluoro-Ether as a bifunctional interphase electrolyte additive with graphite/$LiNi_{0.5}Co_{0.2}Mn_{0.3}O_2$ full cell. *ACS Applied Energy Mater.***2**, 6404-6416 (2019).
7. Ryu H.-H., *et al.* A highly stabilized Ni-rich NCA cathode for high-energy lithium-ion batteries. *Mater. Today* **36**, 73-82 (2020).
8. Kim U.-H., Kuo L.-Y., Kaghazchi P., Yoon C.S. & Sun Y.-K. Quaternary layered Ni-rich NCMA cathode for lithium-ion batteries. *ACS Energy Lett.* **4**, 576-582 (2019).
9. Vidal Laveda J. *et al.* Stabilizing capacity retention in NMC811/graphite full cells *via* TMSPi electrolyte additives. *ACS Applied Energy Mater.* **2**, 7036-7044 (2019).
10. Yu F.-D. *et al.* Dual conductive surface engineering of Li-rich oxides cathode for superior high-energy-density Li-ion batteries. *Nano Energy* **59**, 527-536 (2019).
11. Nayak P.K., Penki T.R., Markovsky B., Aurbach D. Electrochemical performance of Li- and Mn-rich cathodes in full cells with prelithiated graphite negative electrodes. *ACS Energy Lett.* **2**, 544-548 (2017).
12. Yoon M. *et al.* Unveiling nickel chemistry in stabilizing high-voltage cobalt-rich cathodes for lithium-ion batteries. *Adv. Funct. Mater.* **30**, 1907903 (2020).
13. Xu G*, et al.* Tracing the impact of hybrid functional additives on a high-voltage (5 V-class) SiO_x-C/$LiNi_{0.5}Mn_{1.5}O_4$ Li-ion battery system. *Chem. Mater.* **30**, 8291-8302 (2018).
